# Supplementary material for: ‘Giving birth on a beach’: Women’s experiences of using virtual reality in labour
Source: PLoS One. 2024 Jun 12;19(6):e0304349. doi: 10.1371/journal.pone.0304349 (PMC11168694; doi:10.1371/journal.pone.0304349)
Supplement: Attachment — Submitted filename: reviwer comment PONE-D-23-33730.docx [file pone.0304349.s001.docx]

General comment:

I would like to congratulate the authors with the attempt to close the gap in previous research done on this subject. An intersting subject in the world of ever-changing technology and possibilities.

I do however have a number of comments, mainly with the intention of being more precise on how this study closes the gap intended, and how coming research should proceed from here.

A few comments on how to help the reader unfamiliar with the subject, to find further information.

Line 100-102: reference

Line 110-114: 25 included : describe 14 out of 25, 19 out of 25. The 19 out of 25 are described in the methods section, the 25 have been described elsewhere (citation needed) – the 14 out of the 25 needs explanation. This section needs clarification.

114-115: reference – where has it been published

127: Oculus Go: cite manufacturer, link to platform. Help the reader to find the software if interested.

138: possible contraindications to VR use needs clarification. Important for readers that are not familiar to VCR

Line 355: is it viable to use VCR in water or should this be discouraged even though they may be waterproof (line 473) : the problem seemed to be weight as well?

Line 356: are consumer ready products suitable for this specific function – relaxation during labour – or should the be more suitable products – this having been shown in previous studies (lines 466- 470) – can you be more specific into which research needs to be done apart from time ?

Any description of which scenes / music the included participants preferred or not preferred, in general – during the different stages of labour. For example: “I feel it did [have an effect on relaxation], depending on what the scene was” line 301

“For example, Liz synchronised her breathing with the visualisation and sound of the bubbles in the Dolphin” line 307

The term “many women” / “most women” are very general terms. Be more precise: how many (numbers or percentage)

Any differences between women giving birth for the first time and those having given birth previously?

The conclusion is very positive – 94 % of the participants would use it again ( these percentages have not been mentioned before), however challenges have been described. The challenges need to be discussed in the discussion as these challenges need to be addressed in further research

There are a number of well-chosen references in the introduction. How does the present study compare to previous studies: does the study support previous findings, does the present study close the gap intended or are further studies needs if yes – in which direction should these studies be directed, if not – why not

Are there any other aspects of being in labour that may influence the use of VCR?

Does VCR appeal to all women in labour? Table 1 in mind.
