# Supplementary material for: ‘Giving birth on a beach’: Women’s experiences of using virtual reality in labour
Source: PLoS One. 2024 Jun 12;19(6):e0304349. doi: 10.1371/journal.pone.0304349 (PMC11168694; doi:10.1371/journal.pone.0304349)
Supplement: Attachment — Submitted filename: Response to Reviewers.docx [file pone.0304349.s002.docx]

**Response to Reviewers**

Line 100-102: reference – **this has been done in the revised version.**

Line 110-114: 25 included: describe 14 out of 25, 19 out of 25. The 25 have been described elsewhere (citation needed). **This has been done.**

The 14 out of the 25 needs explanation. **This has been done (see tracked changes).**

114-115: reference – where has it been published. **This has been done.**

127: Oculus Go – cite manufacturer – **This has been done.** Link to platform. Help the reader to find the software if interested. **This has been done (see tracked changes).**

138: possible contraindications to VR use. Needs clarification. Important for readers that are not familiar to VR. **This has been done (see tracked changes).**

Line 355: is it viable to use VR in water or should this be discouraged even though they may be waterproof (line 473). The problem seemed to be weight as well. **I think VR in water is very viable as indicated from the women’s narratives. It would be an idea combination – VR and the analgesic effect of water.**

Line 356: are consumer ready products suitable for this specific function – relaxation during labour or should there be more suitable products – this having been shown in previous studies (lines 466 – 470) – can you be more specific into which research needs to be done apart from time. **I have expanded on this (see tracked changes).**

Any descriptions of which scene/music the involved participants preferred or not preferred, in general – during the different stages of labour. For example: “I feel it did [have an effect on relaxation’, depending on what the scene was – line 301.

“For example, Liz synchronised her breathing with the visualisation and sound of the bubbles in the dolphin VE.” Line 307.

**These quotes identify aspects of the scenes that these women preferred, however the preferences were too subjective. No real themes emerged on what scenes were preferred or not preferred. Perhaps if there were more virtual environments available (which there are now).**

The term “many women/”most women” are very general terms. Be more precise: how many (numbers or percentages). **This has been changed (see tracked changes).**

Any difference between women giving birth for the first time and those having given birth previously. **There was no difference in their experiences of using the VR. Some of the women who had had a previous baby were very motivated to try for a non-pharmacological birth based on their previous experience with epidural analgesia.**

The conclusion is very positive, however challenges have been described. The challenges need to be discussed in the discussion as these challenges need to be addressed in future research. **This has been done (see tracked changes).**

There are a number of well-chosen references in the introduction. How does, the present study compare to previous studies: does the study support previous findings, does the present study close the gap it intended or are further studies needed. If yes, which direction should these studies be directed, if not – why not? **This has been done (see tracked changes).**

Are there any other aspects of being in labour that may influence the use of VR? **I can’t see any other aspects.**

Does VR appeal to all women in labour? **I think VR could appeal to all women, in particular young women as they are used to technology. However, results from our study found that it appealed to women who were most motivated to have a non-pharmacological birth. However, I think that there is potential for VR to be used in women have induction of labours who are restricted in movement due to being monitored, IV fluids etc. However, most research studies have as their participants low risk uncomplicated pregnancies and births, but I think future research could be conducted on women with low risk induction of labours.**
