# Supplementary material for: ‘Giving birth on a beach’: Women’s experiences of using virtual reality in labour
Source: PLoS One. 2024 Jun 12;19(6):e0304349. doi: 10.1371/journal.pone.0304349 (PMC11168694; doi:10.1371/journal.pone.0304349)
Supplement: Attachment — Submitted filename: reviewercomments1.docx [file pone.0304349.s004.docx]

**Response to Reviewers**

Thank you to both reviewers for taking the time to review my manuscript and suggest changes.

I am unsure how to proceed given that question 5: Is the manuscript presented in an intelligible fashion and written in standard English.

Reviewer #1: No.

Reviewer #2: Yes

I would argue and agree with reviewer 2 that the manuscript is presented in an intelligible fashion and written in standard English.

6. Review comments to the Author

I have made all the changes requested by Reviewer #1 and they are highlighted as Tracked Changes.

Thank you again
