# Supplementary material for: ‘Giving birth on a beach’: Women’s experiences of using virtual reality in labour
Source: PLoS One. 2024 Jun 12;19(6):e0304349. doi: 10.1371/journal.pone.0304349 (PMC11168694; doi:10.1371/journal.pone.0304349)
Supplement: Attachment — Submitted filename: Reviewer comments.docx [file pone.0304349.s003.docx]

Reviewer comments

Giving Birth on a Beach' Women's Experiences of Using Virtual Reality in Labour PONE-D-23-33730R1

I would like to congratulate the authors on their work in this interesting field which may be at contribution to the implementation and improvement of VCR in the future.

No further comments
